# Research progress on the role and mechanism of circular RNA in drug resistance of head and neck squamous cell carcinoma

**DOI:** 10.20517/cdr.2024.57

**Published:** 2024-09-02

**Authors:** Hongli Zeng, Junshang Ge, Yi Meng, Qian Wang, Mei Yang, Zhaoyang Zeng, Wei Xiong, Xuyu Zu

**Affiliations:** ^1^The First Affiliated Hospital of University of South China, Hengyang 421001, Hunan, China.; ^2^NHC Key Laboratory of Carcinogenesis, Cancer Research Institute, Central South University, Changsha 410078, Hunan, China.

**Keywords:** Head and neck squamous cell carcinoma (HNSCC), circular RNAs (circRNAs), drug resistance

## Abstract

Drug resistance in tumors constitutes a significant obstacle to tumor therapy. Head and neck squamous cell carcinoma (HNSCC) presents a major challenge due to its deep anatomical location, limited space, and complex structure. These factors complicate surgical procedures and hinder the effectiveness of chemoradiotherapy, leading to poor prognosis and reduced quality of life. However, there is hope in the form of circular RNAs (circRNAs), non-coding RNA molecules with a closed-loop structure that exhibits superior stability and resistance to degradation compared to linear RNAs. Recent advances in high-throughput sequencing and bioinformatics technology revealed that circRNAs participate in tumor proliferation, invasion, migration, and drug resistance. This review aims to summarize current research progress on the involvement of circRNAs in drug resistance of HNSCC and provide valuable insights for the prevention and mitigation of drug resistance in HNSCC.

## INTRODUCTION

Head and neck squamous cell carcinoma (HNSCC) originates from the epithelial cells in the head and neck region, encompassing various subtypes, such as oral squamous cell carcinoma (OSCC), laryngeal squamous cell carcinoma (LSCC), nasopharyngeal carcinoma (NPC), and others. HNSCC is the sixth most common cancer worldwide, with more than 890,000 new cases diagnosed each year. Despite advances in treatment, the five-year survival rate has stagnated at around 65% for decades^[1]^. Early detection of HNSCC is challenging due to its deep anatomic location and complex structure, leading to a frequent diagnosis in the middle and late stages of the disease. Consequently, postoperative adjuvant chemotherapy and radiotherapy are often necessary^[2]^. However, drug resistance and radioresistance significantly impact the prognosis of patients with advanced HNSCC.

circRNAs are a unique group of non-coding RNAs that form closed loops due to covalent bonds, therefore lacking 5’ and 3’ ends^[3]^. This unique structure makes circRNAs insensitive to ribonucleases such as exonucleases, ensuring their stability within cells^[4]^. Recent advances in high-throughput sequencing and computational analysis have unveiled the regulatory roles of circRNAs in gene expression in cancer at both pre- and post-transcriptional levels^[5]^. A better understanding of the association between circRNAs and HNSCC will help us to more effectively study and explore new early diagnostic indicators and therapeutic targets for HNSCC. This review aims to clarify the role of circRNAs in drug resistance in HNSCC and its potential as a diagnostic and therapeutic target.

## THE CLINICAL DIAGNOSTIC VALUE OF CIRCRNAS IN HNSCC

circRNAs are abundantly present in cells and play diverse roles in biological processes. Compared to other non-coding RNAs (ncRNAs), such as micro RNAs (miRNAs) and long non-coding RNAs (lncRNAs), circRNAs are more stable in cells due to their closed circular RNA molecular structure. They can function as miRNA sponges, interact with RNA-binding proteins, or be translated into peptides to affect downstream gene expression. Notably, circRNAs often act as “miRNA sponges” or competitive endogenous RNAs (ceRNAs) by containing miRNA binding sites, thereby regulating the function of miRNAs. Additionally, circRNAs can interact with proteins, DNA, RNA, or transcription factors to regulate protein function or gene transcription^[6]^. circRNAs can also encode small peptides and participate in cellular biological processes^[7]^. Furthermore, circRNAs can interact with molecules associated with epigenetic modifications^[8]^, affecting the regulation of various biological processes including tumor cell proliferation, apoptosis, cell differentiation, development and the immune response.

circRNAs possess characteristics such as abundance, ubiquity, conservation, and stable expression in saliva, blood, and exosomes^[9-11]^. Their expression in tissues and organs is stage-specific, making circRNAs highly promising biomarkers. It has been widely proposed that circRNAs could serve as biomarkers for early diagnosis and prognosis of tumors. The development of high-throughput sequencing technologies has facilitated the identification of a growing number of dysregulated circRNAs and their expression patterns are closely associated with patient prognosis. For example, circMAN1A2 exhibits high levels of expression in NPC cell lines and is also significantly increased in the serum of individuals with NPC, indicating its potential as a promising biomarker for the early detection of NPC^[12]^. Similarly, circMYC expression in exosomes can be used to differentiate radiation sensitivity in NPC patients^[13]^, whereas the upregulation of circSERPINA3 is correlated with NPC progression, including lymph node metastasis and unfavorable overall survival outcomes^[14]^. Additionally, high expression of circCRIM1^[15]^ and circ-001387^[16]^ are strongly associated with unfavorable prognosis in patients with NPC. These findings support the potential application of circRNAs in early diagnosis and prognostic evaluation of NPC.

As for OSCC, there remains a deficiency in readily available, precise, and non-invasive biomarkers for early detection. Saliva samples are not only easy to obtain but also rich in circRNAs in OSCC. Certain circRNAs have been observed to exhibit notable upregulation in OSCC cell lines, such as the case of circ-1001242^[17]^ and circ-0086414^[18]^. Furthermore, circ-0001874 and circ-0001971 can be identified in saliva samples from individuals with oral cancer and their expression is strongly linked to the malignancy of the tumors^[19]^. The expression levels of these circRNAs are correlated with tumor stage and size and may be used for the diagnosis of OSCC.

Regarding LSCC, there have also been reports on circRNAs as clinical diagnostic markers. Kan *et al*. examined the whole circRNA expression in LSCC tissues compared with adjacent non-tumor tissues using microarray analysis. They identified 698 circRNAs with differential expressions in LSCC tissues^[20]^. Subsequent quantitative reverse transcription polymerase chain reaction (qRT-PCR) analysis showed the highest upregulation of hsa-circRNA-1008555 and downregulation of hsa-circRNA-1044912 in LSCC. The expression of hsa-circRNA-1008555 was also significantly elevated in LSCC patients who have neck lymph node metastases or are at advanced clinical stages. Conversely, hsa-circRNA-1044912 is significantly decreased in LSCC patients with T3-4 stages, neck lymph node metastases, other advanced clinical stages or poor differentiation^[20]^. These data suggest the correlation between circRNAs and the staging and prognosis of LSCC. Taken together, the identification of circRNAs has great potential for disease diagnosis and prediction of HNSCC.

## THE VALUE OF CIRCRNAS IN THE PROGRESSION OF HNSCC

circRNAs play a significant role in regulating proliferation in HNSCC. Various circRNAs have been identified to promote or inhibit proliferation by interacting with specific miRNAs and modulating downstream target genes. For example, circ-0046263 promotes NPC proliferation by upregulating insulin-like growth factor binding protein 3 (IGFBP3) expression through miR-133a-5p sponge activity^[21]^. circZNF609^[22-24]^, circTMTC1^[25]^, circTRAF3^[26]^, and Epstein-Barr virus (EBV)-encoded circRNA circRPMS1^[27]^ also promote NPC proliferation through distinct mechanisms. Conversely, circKIAA0368^[28]^, circ-0004788^[29]^, circ-0081534^[30,31]^, circITCH^[32]^, circHIPK3^[33]^, and circSETD3^[34,35]^ have been found to inhibit NPC proliferation. In OSCC, circHIPK3^[36]^, circDOCK1^[37]^, hsa-circ-0001971, hsa-circ-0001874^[38]^, and hsa-circ-0011946^[39]^ are overexpressed and stimulate proliferation, whereas hsa-circ-0004491^[40]^, hsa-circ-0002203^[41]^, hsa-circ-0063772^[42]^, circ-0000140^[43]^, hsa-circ-0007059^[44]^, hsa-circ-0055538^[45]^, and hsa-circ-0092125^[46]^ are tumor suppressors that inhibit proliferation and promote apoptosis. circ-100290 functions as a ceRNA by sequestering miR-29b and miR-378a, regulating cyclin-dependent kinase 6 (CDK6) and glucose transporter 1 (GLUT1) expression in OSCC^[47,48]^. circPKD2 promotes apoptosis and inhibits proliferation and invasion in OSCC^[49,50]^. In LSCC, circ-100290 is significantly elevated and enhances proliferation by modulating miR-29a-3p expression^[51]^. Further studies are needed to elucidate the specific regulatory mechanisms of these circRNAs, which could provide potential new targets and therapeutic approaches for HNSCC.

Furthermore, various circRNAs have been identified to promote or inhibit invasion and metastasis of HNSCC by regulating different molecular pathways. For example, circCTDP1^[52]^, circARHGAP12^[53]^, circRNF13^[54]^, circ-0000215^[55]^, circSETD3^[35]^, and circCAMSAP1^[56]^ enhance NPC invasion and metastasis, whereas circCCNB^[57]^, hsa-circ-0000345^[58]^ and circTGFBR2^[59]^ inhibit the migration and invasion of NPC. The overexpression of circPVT1 in NPC significantly promotes NPC migration and invasion, showing a positive correlation with the disease progression and TNM stages of NPC^[60]^. circRILPL1 is highly expressed in NPC and promotes NPC proliferation and metastasis through binding to Rho-associated coiled-coil containing protein kinase 1 (ROCK1) and importin7 (IPO7) to activate the Hippo-YAP signaling pathway^[61]^. In OSCC, circ-0000140^[43]^ and circ-100290^[47]^ promote migration and invasion, whereas circ-0001971^[19,62,63]^ regulates various biological processes. In LSCC, circSHKBP1^[64]^, circ-103862^[65]^, circ-0036722^[66]^, and circBFAR^[67]^ promote proliferation and invasion, whereas circPARD3^[68]^ and circeMTCL1^[69]^ play a role in invasion and migration. Furthermore, several other circRNAs, including circ-0005033^[70]^, circ-0044520^[71]^, and circ-0023028^[72]^, have also been implicated in the regulation of LSCC proliferation, invasion, and migration. These circRNAs interact with specific miRNAs and downstream target genes to modulate the metastatic potential of HNSCC. Further studies are necessary to deepen our understanding of the regulatory mechanisms of these circRNAs and identify potential therapeutic targets for interventions in HNSCC metastasis [Table 1].

**Table 1:** circRNAs associated with proliferation, invasion, and metastasis in HNSCC

**circRNA**	**Expression**	**Functions**	**Targets**	**Tumor**	**Ref.**
circITCH	Downregulation	Proliferation;metastasis; invasion	miR-214	NPC	[32]
circRNF13	Downregulation	Proliferation;metastasis	SUMO2		[54]
circCCNB1	Downregulation	Metastasis; invasion	Nuclear factor 90		[57]
hsa-circ-0000345	Downregulation	Proliferation; metastasis; invasion	miR-513a-3p		[58]
circTGFBR2	Downregulation	Proliferation; metastasis	miR-107		[59]
circ-0046263	Upregulation	Proliferation; metastasis	miR-133a-5p		[21]
circZNF609	Upregulation	Proliferation; metastasis; invasion; angiogenesis	miR-188; miR-150-5p; miR-145		[22-24]
circTRAF3	Upregulation	Proliferation; metastasis	miR-203a-3p		[26]
circRPMS1	Upregulation	Proliferation; invasion; EMT	miR-203; miR-31; miR-451		[27]
circKIAA0368	Upregulation	Proliferation; metastasis; invasion; EMT	miR-6838p-5p		[28]
circ-0004788	Upregulation	Proliferation; metastasis; invasion; angiogenesis	miR-515-5p		[29]
circ-0081534	Upregulation	Proliferation; migration; invasion; EMT	miR-874-3p; miR-508-5p		[30,31]
circCTDP1	Upregulation	Proliferation; metastasis; invasion; apoptosis	miR-320b		[52]
circARHGAP12	Upregulation	Metastasis; invasion	EZR; TPM3; RhoA		[53]
circSETD3	Upregulation	Metastasis; invasion; proliferation	miR-615-5; miR-1538; miR-147a		[34,35]
circ-0000215	Upregulation	Proliferation; metastasis	miR-512-5p		[55]
circCAMSAP1	Upregulation	Proliferation; metastasis	SERPINH1		[56]
circTMTC1	Upregulation	Proliferation; migration; invasion; EMT; apoptosis	miR-495		[25]
circHIPK3	Upregulation	Proliferation; metastasis; invasion	miR-4288		[33]
circRILPL1	Upregulation	Proliferation; metastasis	ROCK1; IPO7		[61]
circPVT1	Upregulation	Metastasis; invasion	E3 ubiquiting ligase β-TrCP		[60]
circ-0000140	Downregulation	Proliferation; metastasis; invasion	miR-182-5p	OSCC	[43]
hsacirc0001971/hsa-circ-0001874	Upregulation	Proliferation; apoptosis	miR-194; miR-204; miR-186/miR-296		[38]
circPKD2	Downregulation	Proliferation; metastasis; invasion	miR-204-3p		[49,50]
hsa-circ-0002203	Downregulation	Proliferation; metastasis; invasion; apoptosis	/		[41]
hsa-circ-0004491	Downregulation	Metastasis; invasion	/		[40]
hsa-circ-0007059	Downregulation	Proliferation; metastasis; invasion	AKT/mTOR		[44]
hsa-circ-0055538	Downregulation	Proliferation	p53/Bcl-2/caspase		[45]
hsa-circ-0063772	Downregulation	Proliferation; metastasis; invasion	/		[42]
hsa-circ-0092125	Downregulation	Proliferation; metastasis; invasion	/		[46]
circ-100290	Upregulation	Proliferation; glycolysis	miR-29b; miR-378a		[47,48]
circDOCK1	Upregulation	Proliferation	miR-196a-5p		[37]
circHIPK3	Upregulation	Proliferation; metastasis; invasion; EMT; apoptosis	miR-124; miR-381-3p; miR-637		[36]
hsa-circ-0001971	Upregulation	Proliferation; apoptosis	miR-186; miR-107		[19,62,63]
hsa-circ-0011946	Upregulation	Proliferation; metastasis; invasion	miR3383p		[39]
circPARD3	Downregulation	Proliferation	/	LSCC	[68]
circ-0036722	Downregulation	Proliferation	miR-1248		[66]
circ-100290	Upregulation	Proliferation	miR-29a-3p		[51]
circSHKBP1	Upregulation	Proliferation; invasion	miRNA-766-5p		[64]
circ-0005033	Upregulation	Proliferation; metastasis; invasion	miR-107		[70]
circ-0044520	Upregulation	Proliferation; metastasis; invasion	miR-338-3p		[71]
circ-0023028	Upregulation	Proliferation; metastasis	miR-486-3p		[72]
circ-103862	Upregulation	Proliferation; invasion	miR-493-5p		[65]
circBFAR	Upregulation	Proliferation; invasion	miRNA-31-5p		[67]
cireMTCL1	Upregulation	Proliferation; metastasis; invasion	CIQBP		[69]

circRNAs: Circular RNAs; HNSCC: head and neck squamous cell carcinoma; EMT: Epithelial-Mesenchymal Transition; NPC: nasopharyngeal carcinoma; OSCC: oral squamous cell carcinoma; LSCC: laryngeal squamous cell carcinoma.

## ROLE OF CIRCRNAS IN RADIOTHERAPY, CHEMOTHERAPY, AND IMMUNOTHERAPY OF HNSCC

### circRNAs are associated with radioresistance in HNSCC

Radiotherapy resistance is a significant challenge in the treatment of HNSCC and is closely related to patient prognosis. circRNAs are involved in this process. For example, overexpression of circ-000543 in radioinsensitive NPC contributes to the development of radioresistance. This circRNA functions by binding to miR-9, thereby upregulating platelet-derived growth factor receptor B (PDGFRB) expression^[73]^.

In addition, circRNAs can also modulate genes involved in the DNA damage response and repair. For example, the expression of circATRNL1 significantly decreased after radiation exposure, which may lead to radiation resistance in tumor cells by inhibiting apoptosis and cell cycle arrest in OSCC. Therefore, increasing the expression of circATRNL1 may help improve the sensitivity of OSCC to radiotherapy, thereby enhancing its effectiveness^[74]^. In general, circRNAs affect the responsiveness of tumors to radiotherapy by regulating apoptosis pathways and DNA damage response and repair pathways post-radiation. Thus, identifying targets for radiosensitization and reversing radioresistance of HNSCC is of great interest.

### circRNAs are associated with immune evasion in HNSCC

circRNAs can regulate the malignant progression of head and neck cancer through mechanisms such as immune evasion^[75]^. This mechanism refers to the escape of tumor cells from the immune system attack through different mechanisms, thus promoting tumor proliferation and invasion. Immune evasion mechanisms include inhibiting the activity of immune cells, changing tumor cell surface molecules to avoid being recognized by the immune system, and producing immunosuppressive factors, among others. By studying the mechanism of immune evasion, researchers found that the process can be regulated to achieve therapeutic effects such as inhibiting tumor proliferation and invasion. Immunotherapy has been widely used in HNSCC. Immune checkpoint inhibitors, in particular, are a common form of immunotherapy^[76]^. These drugs enhance the ability of immune cells to attack tumors by blocking the inhibitory signals between tumor cells and immune cells. In addition, personalized immunotherapy is also used in HNSCC. This therapy analyzes the patient’s tumor tissue or blood samples, identifies the tumor-specific antigens, and uses those antigens to activate the patient’s own immune system to attack and kill the tumor cells^[77]^.

An example of a circRNA associated with immune evasion in HNSCC is circBART2.2, encoded by EBV, which can promote programmed death-ligand 1 (PD-L1) expression by activating the innate immune signaling pathway, leading to immune escape in NPC^[78]^. In addition, circCDR1as can regulate the immune escape process of OSCC by regulating the expression of miR-7^[79]^. Similarly, has-circ-0069313 participates in the immune escape of OSCC by modulating the expression of miR-325-3p and subsequently affecting forkhead box P3 (FOXP3)^[80]^. These circRNAs may influence the immune escape ability of LSCC by modulating immune-related genes.

### circRNAs are associated with drug resistance in HNSCC

Chemotherapy is one of the most effective treatments for advanced HNSCC. Currently, the commonly used first-line chemotherapy drugs include platinum drugs, 5-fluorouracil, imidazoles and taxanes. Platinum drugs such as cisplatin and carboplatin inhibit the growth and division of cancer cells by interfering with DNA replication and repair processes. The drug 5-fluorouracil (5-FU) is an antimetabolite drug that blocks the growth and division of cancer cells by inhibiting DNA and RNA synthesis^[81]^. Imidazoles, such as methotrexate, also inhibit the growth and division of cancer cells by blocking DNA and RNA synthesis^[82]^. Taxanes, such as paclitaxel and docetaxel, block the division and spread of cancer cells by interfering with microtubule polymerization^[83]^. Overall, research on chemotherapy for HNSCC is progressing with the hope of finding more effective treatments to improve the patients’ survival rate and quality of life. However, a significant number of HNSCC patients exhibit chemotherapy resistance, posing a major challenge to effective treatment.

**Figure 1:**
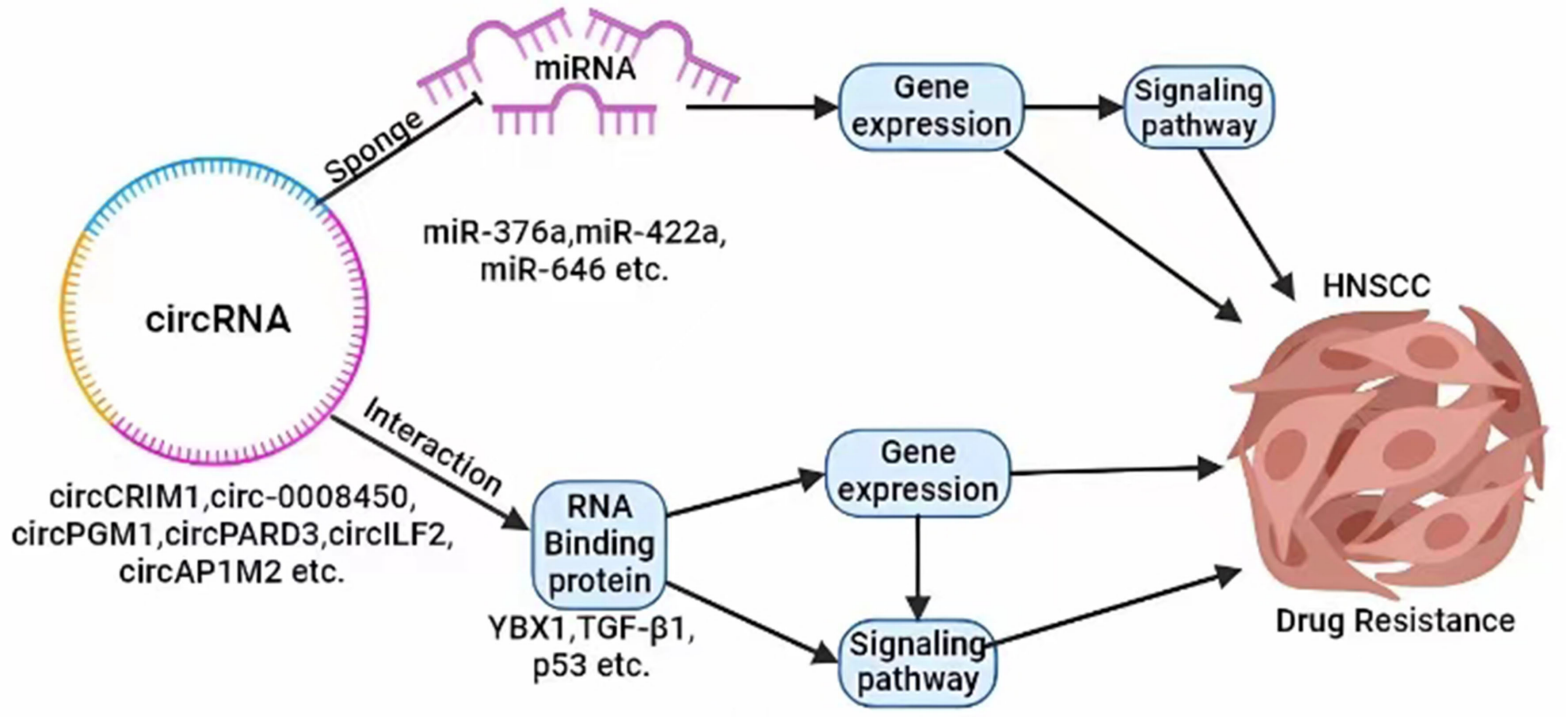
circRNAs are closely associated with signaling pathways involved in drug resistance of HNSCC. circRNAs: Circular RNAs; HNSCC: head and neck squamous cell carcinoma.

The emergence of multidrug resistance in tumor cells is the main reason for chemotherapy failure^[84,85]^. With the extensive investigation of circRNA biological function, increasing evidence suggests that certain circRNAs are significantly upregulated in drug-resistant HNSCC tissues. Furthermore, circRNAs are closely associated with some signaling pathways that are involved in drug resistance of HNSCC, such as PI3K/AKT, MAPK, NF-κB, and Wnt/β-catenin signaling pathways^[86]^. These circRNAs can act as "sponges" for miRNAs by containing multiple miRNA binding sites, thereby adsorbing and inhibiting miRNA activity and subsequently regulating downstream pathways that affect tumor sensitivity to drugs. Additionally, circRNAs can influence tumor drug resistance, either by directly binding to proteins or through the regulation of cellular autophagy^[75]^ [Figure 1].

#### circRNAs and chemoresistance in nasopharyngeal carcinoma

Specific circRNAs have been reported to play a role in metastasis and resistance to chemotherapy in NPC. For instance, circCRIM1 is abundantly expressed in NPC tissues with high metastasis and can enhance forkhead box Q1 (FOXQ1) expression by targeting miR-422a. This, in turn, promotes NPC metastasis and confers resistance to docetaxel chemotherapy^[87]^. circ-0008450 is upregulated in NPC tissues and promotes resistance to cisplatin by targeting the miR-338-3p/Smad5 axis^[88]^. circIPO7 is upregulated in NPC tissues and binds to Y-box binding protein1 (YBX1) in the cytoplasm, promoting its phosphorylation at serine102 (p-YBX1S102) by the kinase AKT. This leads to the nuclear translocation of YBX1 and activation of genes such as fibroblast growth factor receptor 1 (FGFR1), tenascin-C (TNC) and neurotrophic tyrosine kinase receptor 1 (NTRK1), thereby promoting resistance to cisplatin in NPC^[89]^. Another example is circ-0067717, which is upregulated in paclitaxel-resistant NPC. This circRNA serves as a scaffold of tripartite motif containing 41 (TRIM41) protein, which is a ubiquitin E3 ligase. TRIM41 induces p53 ubiquitination and degradation, which results in reduced levels of p53 protein, ultimately promoting resistance to paclitaxel in NPC^[90]^. circPARD3 promotes cisplatin resistance of NPC side population cells. This effect is mediated through the miR-579-3p/SIRT1/SSRP1 axis^[91]^. circ-0028007 derived from the *NUAK1* gene is upregulated in poorly differentiated NPC cell lines and its silencing enhances the responsiveness of NPC to paclitaxel/cisplatin^[92]^. circNRIP1 is upregulated in the serum of chemotherapy-resistant NPC cells, and functions as a sponge of miR-515-5p, leading to miR-515-5p inhibition and interleukin 25 (IL-25) upregulation. This mechanism promotes chemotherapy resistance in NPC by enhancing the expression of IL-25, which can be directly targeted by miR-515-5p^[93]^. circSETD3 is upregulated in NPC and is related to cisplatin resistance in this carcinoma. Knockdown of circSETD3 can inhibit NPC proliferation, enhance cisplatin sensitivity, and increase the apoptosis rate. circSETD3, a sponge for miR-147a, promotes activation of the AKT/mTOR pathway. circSETD3 promotes NPC proliferation and cisplatin resistance by manipulating miR-147a^[34]^.

#### circRNAs and chemoresistance of laryngeal squamous cell carcinoma

circRNAs affect the chemotherapy resistance of LSCC. For example, circPGM1 interacts with miR-376a as a ceRNA to regulate the drug resistance of LSCC. The autophagy-related protein 2A (ATG2A) is a key effector of circPGAM1/miR-376a axis-mediated resistance. Overexpression of ATG2A significantly inhibits miR-376a on cisplatin resistance in LSCC. circPGAM1 functions as a miR-376a sponge, promoting the expression of ATG2A and subsequently enhancing autophagy, leading to cisplatin resistance in LSCC^[94]^. Gao *et al*. observed a significant association between elevated circPARD3 expression and tumor stages and cervical lymph node metastasis of LSCC patients^[68]^. Moreover, high circPARD3 caused significantly shorter overall survival of LSCC patients. circPARD3 functions as a miRNA-145-5p sponge, upregulating the expression of protein kinase C iota (PRKCI) and further activating the AKT/mTOR signaling axis, promoting the proliferation, migration, invasion and chemotherapy resistance of LSCC^[68]^. Yi *et al*. observed elevated expression of circ-0004507 in LSCC and noted a positive correlation between its expression and tumor stage, differentiation degree, and lymph node metastasis rate^[95]^. circ-0004507 was highly expressed in the cancer tissues of chemotherapy-resistant patients. Through its role as a miR-873 sponge, circ-0004507 downregulates the expression of miR-873, thereby reducing the sensitivity of laryngeal cancer to cisplatin chemotherapy^[95]^. Additionally, Gong *et al*. showed that circ-0005033 was highly expressed in laryngeal cancer tissues, compared with normal laryngeal epithelial tissues, and circ-0005033 functions as a ceRNA for miR-107 in LSCC^[70]^. High expression of circ-0005033 downregulates miRNA-107 expression, promoting insulin-like growth factor-1 receptor (IGF1R) expression and subsequently enhancing the proliferation, invasion, migration, and cisplatin resistance of LSCC^[70]^**.**

#### circRNAs and chemoresistance in oralsquamous cell carcinoma

Some circRNAs show significant differential expression between chemotherapy-resistant OSCC patients and corresponding normal tissues. Wu *et al*. discovered that circILF2 is overexpressed in cisplatin-resistant OSCC cells^[96]^. As a miRNA sponge for miR-1252, circ-ILF2 inhibits miR-1252 expression. miR-1252 regulates the expression of Krüppel-like factor 8 (KLF8), which functions on proliferation, drug resistance, and inflammation of OSCC, thereby promoting cisplatin resistance^[96]^. The researchers identified circAP1M2 (hsa-circ-0049282) as a miR-1249-3p sponge, inhibiting autophagy-related protein 9A (9A) expression. circAP1M2 activates autophagy associated with ATG9A, thereby promoting cisplatin resistance in OSCC^[97]^. circ-0109291 is also overexpressed in cisplatin-resistant OSCC and promotes proliferation and cisplatin resistance of OSCC cells through the miR-188-3p/ABCB1 axis^[98]^. The circRNA circANKS1B (circ-0007294), originating from exons 5 to 8 of the *ANKS1B* gene, is positively associated with the expression of transforming growth factor-β1 (TGF-β1) in OSCC tissues. circANKS1B facilitates the growth and resistance of OSCC by stimulating the TGF-β signaling pathway in oral cancer cells^[99]^. circPKD2 is significantly upregulated in cisplatin-treated OSCC and acts as a miR-646 sponge in OSCC cells. By inhibiting miR-646, circPKD2 promotes the expression of autophagy-related protein 13 (ATG13), leading to the accumulation of autophagic vesicles in cisplatin-treated OSCC cells and enhancing sensitivity to cisplatin^[49]^ [Table 2].

**Table 2:** circRNAs involvement in chemotherapy resistance of HNSCC

**circRNA**	**Tumor**	**Expression**	**Functions**	**Targets**	**Ref.**
circCRIM1	NPC	Upregulation	Promote metastasis of NPC and resistance to docetaxel chemotherapy	miR-422a	[87]
circ-0008450		Upregulation	Promotes NPC cisplatin resistance	miR-338-3p	[88]
circIPO7		Upregulation	Promotes NPC cells to resist DNA damage triggered by cisplatin	YBX1	[89]
circ-0067717		Upregulation	Promote taxol resistance in NPC	TRIM41; p53	[90]
circPARD3		Upregulation	Promote the dryness and cisplatin resistance of NPC	miR-579-3p	[91]
circ-0028007		Upregulation	Decreased sensitivity of NPC to paclitaxel/cisplatin	\	[92]
circNRIP1		Upregulation	Promote NPC drug resistance	miR-515-5p	[93]
circSETD3		Upregulation	Promotes NPC cisplatin resistance	miR-147a	[34]
circPGM1	LSCC	\	Promote cisplatin resistance in LSCC cells	miR-376a	[94]
circPARD3		Upregulation	Promote the proliferation, migration, invasion and chemotherapy resistance of LSCC	miR-145-5p	[68]
circ-0004507		Upregulation	Reduce the sensitivity of LSCC to cisplatin chemotherapy	miR-873	[95]
circ-0005033		Upregulation	Promote proliferation, invasion, migration and cisplatin resistance of LSCC	miR-107	[70]
circ-ILF2	OSCC	Upregulation	Promote OSCC of cisplatin resistance cell carcinoma	miR-1252	[96]
circAP1M2		Upregulation	Promotes autophagy associated cisplatin resistance in OSCC	miR-1249-3p	[97]
circ-0109291		Upregulation	Promote the proliferation and cisplatin resistance of intracavitary squamous cell cells	miR-188-3p	[98]
circANKS1B		Upregulation	Promote the growth and drug resistance of intracavitary squamous cell carcinoma	TGF-β1	[99]
circPKD2		Upregulation	Promoting cisplatin sensitivity in OSCC	miR-646	[49]

circRNAs: Circular RNAs; HNSCC: head and neck squamous cell carcinoma; NPC: nasopharyngeal carcinoma; LSCC: laryngeal squamous cell carcinoma; OSCC: oral squamous cell carcinoma.

In conclusion, circRNAs have emerged as promising biomarkers for various cancers, including NPC, OSCC, and LSCC. Their dysregulation affects the development and progression of these malignancies. By further investigating the functional roles and underlying mechanisms of circRNAs in these cancers, we can potentially discover new diagnostic and therapeutic targets. Therefore, circRNAs have significant potential for enhancing the management and prognosis of patients with NPC, OSCC, and LSCC. Further research in this area is necessary to fully comprehend circRNAs’ clinical implications and therapeutic uses in these specific cancer types.

## PERSPECTIVES

Overall, circRNA has great potential for clinical application in HNSCC. Firstly, circRNA can serve as a potential biomarker for HNSCC^[51,72]^. Detecting the expression level of circRNA can aid in the early diagnosis of HNSCC, evaluating the prognosis of patients and guiding the selection of individualized treatment options. Secondly, circRNA can be used as a predictor of drug resistance in HNSCC^[68]^. By examining the expression level of circRNA, patients’ sensitivity to chemotherapeutic agents can be predicted, thus guiding the choice of individualized treatment options^[9,18]^. Further investigation of the mechanism of circRNA in drug resistance in HNSCC could provide important clues for discovering novel therapeutic targets.

However, current research on circRNA is still in its early stages. Most studies focus on phenotype observation and molecular regulation, with many studies on circRNA mechanisms remaining at the level of comparative expression profile construction following high-throughput sequencing. Although bioinformatics analysis methods have been used to predict the biological functions and signaling pathways of many circRNAs, comprehensive studies on the mechanisms of multiple signaling pathways are still lacking, especially in clinical applications. To further improve the effectiveness of tumor treatment, new targets and molecular biomarkers need to be discovered and identified. Additionally, the mechanisms underlying the occurrence and development of HNSCC have not been fully clarified and the sample size of clinical correlation research is small, limiting its translational value.

Although current research on circRNAs and their functions has uncovered only the tip of the iceberg, advances in research methods and technologies will eventually unveil the full scope of their significance. circRNAs will become increasingly important in the diagnosis and treatment of HNSCC. As research progresses, more chemoresistance regimens for HNSCC will be developed and applied clinically. The connection between circRNAs and HNSCC treatment will become more prominent. At that time, circRNAs may become effective diagnostic and prognostic biomarkers for HNSCC and potentially serve as new targets for treatment^[100]^.

## DECLARATIONS

### Copyright

© The Author(s) 2024.

## References

[1] Johnson DE, Burtness B, Leemans CR, Lui VWY, Bauman JE, Grandis JR (2020). Head and neck squamous cell carcinoma. Nat Rev Dis Primers.

[2] Sacco AG, Cohen EE (2015). Current treatment options for recurrent or metastatic head and neck squamous cell carcinoma. J Clin Oncol.

[3] Beermann J, Piccoli MT, Viereck J, Thum T (2016). Non-coding RNAs in development and disease: background, mechanisms, and therapeutic approaches. Physiol Rev.

[4] Suzuki H, Zuo Y, Wang J, Zhang MQ, Malhotra A, Mayeda A (2006). Characterization of RNase R-digested cellular RNA source that consists of lariat and circular RNAs from pre-mRNA splicing. Nucleic Acids Res.

[5] Harper KL, Mcdonnell E, Whitehouse A (2019). CircRNAs: from anonymity to novel regulators of gene expression in cancer (Review). Int J Oncol.

[6] Chen LL (2020). The expanding regulatory mechanisms and cellular functions of circular RNAs. Nat Rev Mol Cell Biol.

[7] Wu P, Mo Y, Peng M (2020). Emerging role of tumor-related functional peptides encoded by lncRNA and circRNA. Mol Cancer.

[8] Yang Y, Wang Y, Wang F (2020). The roles of miRNA, lncRNA and circRNA in the development of osteoporosis. Biol Res.

[9] Yong-Deok K, Eun-Hyoung J, Yeon-Sun K (2015). Molecular genetic study of novel biomarkers for early diagnosis of oral squamous cell carcinoma. Med Oral Patol Oral Cir Bucal.

[10] Zhang SJ, Chen X, Li CP (2017). Identification and characterization of circular RNAs as a new class of putative biomarkers in diabetes retinopathy. Invest Ophthalmol Vis Sci.

[11] Li Y, Zheng Q, Bao C (2015). Circular RNA is enriched and stable in exosomes: a promising biomarker for cancer diagnosis. Cell Res.

[12] Dang QQ, Li PH, Wang J (2023). CircMAN1A2 contributes to nasopharyngeal carcinoma progression via enhancing the ubiquitination of ATMIN through miR-135a-3p/UBR5 axis. Hum Cell.

[13] Luo Y, Ma J, Liu F, Guo J, Gui R (2020). Diagnostic value of exosomal circMYC in radioresistant nasopharyngeal carcinoma. Head Neck.

[14] Liu R, Zhou M, Zhang P, Zhao Y, Zhang Y (2020). Cell proliferation and invasion is promoted by circSERPINA3 in nasopharyngeal carcinoma by regulating miR-944/MDM2 axis. J Cancer.

[15] He W, Zhou X, Mao Y (2022). CircCRIM1 promotes nasopharyngeal carcinoma progression via the miR-34c-5p/FOSL1 axis. Eur J Med Res.

[16] Shuai M, Huang L (2020). High expression of hsa_circRNA_001387 in nasopharyngeal carcinoma and the effect on efficacy of radiotherapy. Onco Targets Ther.

[17] Sufianov A, Begliarzade S, Kudriashov V (2023). The role of circular RNAs in the pathophysiology of oral squamous cell carcinoma. Noncoding RNA Res.

[18] Li L, Zhang ZT (2020). Hsa_circ_0086414 might be a diagnostic biomarker of oral squamous cell carcinoma. Med Sci Monit.

[19] Zhao SY, Wang J, Ouyang SB, Huang ZK, Liao L (2018). Salivary circular RNAs Hsa_Circ_0001874 and Hsa_Circ_0001971 as novel biomarkers for the diagnosis of oral squamous cell carcinoma. Cell Physiol Biochem.

[20] Kan X, Sun Y, Lu J (2016). Co-inhibition of miRNA-21 and miRNA-221 induces apoptosis by enhancing the p53-mediated expression of pro-apoptotic miRNAs in laryngeal squamous cell carcinoma. Mol Med Rep.

[21] Yin L, Chen J, Ma C (2020). Hsa_circ_0046263 functions as a ceRNA to promote nasopharyngeal carcinoma progression by upregulating IGFBP3. Cell Death Dis.

[22] Li M, Li Y, Yu M (2020). CircRNA ZNF609 knockdown suppresses cell growth via modulating miR-188/ELF2 axis in nasopharyngeal carcinoma. Onco Targets Ther.

[23] Wang J, Lin Y, Jiang DH, Yang X, He XG (2021). CircRNA ZNF609 promotes angiogenesis in nasopharyngeal carcinoma by regulating miR-145/STMN1 axis. Kaohsiung J Med Sci.

[24] Zhu L, Liu Y, Yang Y, Mao XM, Yin ZD (2019). CircRNA ZNF609 promotes growth and metastasis of nasopharyngeal carcinoma by competing with microRNA-150-5p. Eur Rev Med Pharmacol Sci.

[25] Zhao Y, Li C, Zhang Y, Li Z (2022). CircTMTC1 contributes to nasopharyngeal carcinoma progression through targeting miR-495-MET-eIF4G1 translational regulation axis. Cell Death Dis.

[26] Fang X, Huang W, Wu P, Zeng J, Li X (2021). CircRNA circTRAF3 promotes nasopharyngeal carcinoma metastasis through targeting miR-203a-3p/AKT3 axis. Pathol Res Pract.

[27] Liu Q, Shuai M, Xia Y (2019). Knockdown of EBV-encoded circRNA circRPMS1 suppresses nasopharyngeal carcinoma cell proliferation and metastasis through sponging multiple miRNAs. Cancer Manag Res.

[28] Chen Z, Gong Q, Li D, Zhou J (2022). CircKIAA0368 promotes proliferation, migration, and invasion by upregulating HOXA10 in nasopharyngeal carcinoma. Am J Rhinol Allergy.

[29] Li D, Li X, Fan G, Bian G (2022). Identification of the regulatory role of the circ_0004788/miR-515-5p/FGF2 network in nasopharyngeal carcinoma development. Head Neck.

[30] He J, Chen S, Wu X, Jiang D, Li R, Mao Z (2022). Hsa_circ_0081534 facilitates malignant phenotypes by sequestering miR-874-3p and upregulating FMNL3 in nasopharyngeal carcinoma. Auris Nasus Larynx.

[31] Li S, Wang Q (2020). Hsa_circ_0081534 increases the proliferation and invasion of nasopharyngeal carcinoma cells through regulating the miR-508-5p/FN1 axis. Aging.

[32] Liu T, Huang T, Shang M, Han G (2022). CircRNA ITCH: insight into its role and clinical application prospect in tumor and non-tumor diseases. Front Genet.

[33] Ke Z, Xie F, Zheng C, Chen D (2019). CircHIPK3 promotes proliferation and invasion in nasopharyngeal carcinoma by abrogating miR-4288-induced ELF3 inhibition. J Cell Physiol.

[34] Deng G, Wang F, Song Y (2022). Circular RNA SET domain protein 3 promotes nasopharyngeal carcinoma proliferation, cisplatin resistance, and protein kinase B/mammalian target of rapamycin pathway activation by modulating microRNA-147a expression. Bioengineered.

[35] Tang L, Xiong W, Zhang L (2021). circSETD3 regulates MAPRE1 through miR-615-5p and miR-1538 sponges to promote migration and invasion in nasopharyngeal carcinoma. Oncogene.

[36] Jiang W, Zhang C, Zhang X, Sun L, Li J, Zuo J (2021). CircRNA HIPK3 promotes the progression of oral squamous cell carcinoma through upregulation of the NUPR1/PI3K/AKT pathway by sponging miR-637. Ann Transl Med.

[37] Wang L, Wei Y, Yan Y (2018). CircDOCK1 suppresses cell apoptosis via inhibition of miR‑196a‑5p by targeting BIRC3 in OSCC. Oncol Rep.

[38] Jun W, Shaobo O, Xianhua Z (2021). Deregulation of hsa_circ_0001971/miR-186 and hsa_circ_0001874/miR-296 signaling pathways promotes the proliferation of oral squamous carcinoma cells by synergistically activating SHP2/PLK1 signals. Sci Rep.

[39] Meng Y, Zhao EY, Zhou Y (2020). Circular RNA hsa_circ_0011946 promotes cell growth, migration, and invasion of oral squamous cell carcinoma by upregulating PCNA. Eur Rev Med Pharmacol Sci.

[40] Li X, Zhang H, Wang Y, Sun S, Shen Y, Yang H (2019). Silencing circular RNA hsa_circ_0004491 promotes metastasis of oral squamous cell carcinoma. Life Sci.

[41] Su W, Wang YF, Wang F, Yang HJ, Yang HY (2019). [Effect of circular RNA hsa_circ_0002203 on the proliferation, migration, invasion, and apoptosis of oral squamous cell carcinoma cells]. Hua Xi Kou Qiang Yi Xue Za Zhi.

[42] Wang F, Wang YF, Su W, Yang HJ, Yang HY (2019). [Effect of circular RNA hsa_circ_0063772 on proliferation, migration and invasion of oral squamous cell carcinoma cells]. Zhonghua Kou Qiang Yi Xue Za Zhi.

[43] Guo J, Su Y, Zhang M (2020). Circ_0000140 restrains the proliferation, metastasis and glycolysis metabolism of oral squamous cell carcinoma through upregulating CDC73 via sponging miR-182-5p. Cancer Cell Int.

[44] Su W, Wang Y, Wang F (2019). Circular RNA hsa_circ_0007059 indicates prognosis and influences malignant behavior via AKT/mTOR in oral squamous cell carcinoma. J Cell Physiol.

[45] Su W, Sun S, Wang F, Shen Y, Yang H (2023). Retraction note: circular RNA hsa_circ_0055538 regulates the malignant biological behavior of oral squamous cell carcinoma through the p53/Bcl-2/caspase signaling pathway. J Transl Med.

[46] Gao L, Wang QB, Zhi Y (2020). Down-regulation of hsa_circ_0092125 is related to the occurrence and development of oral squamous cell carcinoma. Int J Oral Maxillofac Surg.

[47] Chen L, Zhang S, Wu J (2017). circRNA_100290 plays a role in oral cancer by functioning as a sponge of the miR-29 family. Oncogene.

[48] Chen X, Yu J, Tian H (2019). Circle RNA hsa_circRNA_100290 serves as a ceRNA for miR-378a to regulate oral squamous cell carcinoma cells growth via Glucose transporter-1 (GLUT1) and glycolysis. J Cell Physiol.

[49] Gao L, Zhang Q, Li S, Zheng J, Ren W, Zhi K (2022). Circ-PKD2 promotes Atg13-mediated autophagy by inhibiting miR-646 to increase the sensitivity of cisplatin in oral squamous cell carcinomas. Cell Death Dis.

[50] Gao L, Zhao C, Li S (2019). circ-PKD2 inhibits carcinogenesis via the miR-204-3p/APC2 axis in oral squamous cell carcinoma. Mol Carcinog.

[51] Wang Z, Huang C, Zhang A, Lu C, Liu L (2020). Overexpression of circRNA_100290 promotes the progression of laryngeal squamous cell carcinoma through the miR-136-5p/RAP2C axis. Biomed Pharmacother.

[52] Li H, You J, Xue H, Tan X, Chao C (2020). CircCTDP1 promotes nasopharyngeal carcinoma progression via a microRNA-320b/HOXA10/TGFβ2 pathway. Int J Mol Med.

[53] Fan C, Qu H, Xiong F (2021). CircARHGAP12 promotes nasopharyngeal carcinoma migration and invasion via ezrin-mediated cytoskeletal remodeling. Cancer Lett.

[54] Mo Y, Wang Y, Zhang S (2021). Circular RNA circRNF13 inhibits proliferation and metastasis of nasopharyngeal carcinoma via SUMO2. Mol Cancer.

[55] Chen X, Xu W, Ma Z (2021). Circ_0000215 exerts oncogenic function in nasopharyngeal carcinoma by targeting miR-512-5p. Front Cell Dev Biol.

[56] Wang Y, Yan Q, Mo Y (2022). Splicing factor derived circular RNA circCAMSAP1 accelerates nasopharyngeal carcinoma tumorigenesis via a SERPINH1/c-Myc positive feedback loop. Mol Cancer.

[57] Zhao M, Wang Y, Tan F (2022). Circular RNA circCCNB1 inhibits the migration and invasion of nasopharyngeal carcinoma through binding and stabilizing TJP1 mRNA. Sci China Life Sci.

[58] Jiang C, Li H, Liu F, Shi L, Liu J, Li Y (2022). Hsa_circ_0000345 inhibits cell proliferation, migration and invasion of nasopharyngeal carcinoma cells via miR-513a-3p/PTEN axis. J Physiol Sci.

[59] Li W, Lu H, Wang H (2021). Circular RNA TGFBR2 acts as a ceRNA to suppress nasopharyngeal carcinoma progression by sponging miR-107. Cancer Lett.

[60] Mo Y, Wang Y, Wang Y (2022). Circular RNA circPVT1 promotes nasopharyngeal carcinoma metastasis via the β-TrCP/c-Myc/SRSF1 positive feedback loop. Mol Cancer.

[61] Wu P, Hou X, Peng M (2023). Circular RNA circRILPL1 promotes nasopharyngeal carcinoma malignant progression by activating the Hippo-YAP signaling pathway. Cell Death Differ.

[62] Lu X, Xie H (2023). Circ_0001971 makes progress of oral squamous cell carcinoma by targeting miR-107/FZD4 axis. Oral Dis.

[63] Tan X, Zhou C, Liang Y, Lai YF, Liang Y (2020). Circ_0001971 regulates oral squamous cell carcinoma progression and chemosensitivity by targeting miR-194/miR-204 in vitro and in vivo. Eur Rev Med Pharmacol Sci.

[64] Chen F, Zhang H, Wang J (2022). Circular RNA CircSHKBP1 accelerates the proliferation, invasion, angiogenesis, and stem cell-like properties via modulation of microR-766-5p/high mobility group AT-hook 2 axis in laryngeal squamous cell carcinoma. Bioengineered.

[65] Wang X, Wu T, Wang P (2020). Circular RNA 103862 promotes proliferation and invasion of laryngeal squamous cell carcinoma cells through the miR-493-5p/GOLM1 axis. Front Oncol.

[66] Guo Y, Huang Q, Zheng J (2020). Diagnostic role of dysregulated circular RNA hsa_circ_0036722 in laryngeal squamous cell carcinoma. Onco Targets Ther.

[67] Gong H, Wu W, Fang C, He D (2022). CircBFAR correlates with poor prognosis and promotes laryngeal squamous cell cancer progression through miR-31-5p/COL5A1 axis. Laryngoscope Investig Otolaryngol.

[68] Gao W, Guo H, Niu M (2020). circPARD3 drives malignant progression and chemoresistance of laryngeal squamous cell carcinoma by inhibiting autophagy through the PRKCI-Akt-mTOR pathway. Mol Cancer.

[69] Wang Z, Sun A, Yan A (2022). Circular RNA MTCL1 promotes advanced laryngeal squamous cell carcinoma progression by inhibiting C1QBP ubiquitin degradation and mediating beta-catenin activation. Mol Cancer.

[70] Gong L, Chen J, Jiang X (2022). Circ_0005033 is an oncogene in laryngeal squamous cell carcinoma and regulates cell progression and Cisplatin sensitivity via miR-107/IGF1R axis. Anticancer Drugs.

[71] Yang H, Yu G, Wang Y, Guo X (2022). Circ_0044520 regulates the progression of laryngeal squamous cell carcinoma via the miR-338-3p/ROR2 axis. Histol Histopathol.

[72] Zheng Y, Duan L, Yang Y, Luo D, Yan B (2021). Circ_0023028 contributes to the progression of laryngeal squamous cell carcinoma by upregulating LASP1 through miR-486-3p. Mol Cell Biochem.

[73] Chen L, Zhou H, Guan Z (2019). CircRNA_000543 knockdown sensitizes nasopharyngeal carcinoma to irradiation by targeting miR-9/platelet-derived growth factor receptor B axis. Biochem Biophys Res Commun.

[74] Zhu J, Fu Q, Shao J (2021). Regulating effect of Circ_ATRNL1 on the promotion of SOX9 expression to promote chondrogenic differentiation of hAMSCs mediated by MiR-145-5p. J Tissue Eng Regen Med.

[75] Han X, Tian R, Wang C, Li Y, Song X (2022). CircRNAs: roles in regulating head and neck squamous cell carcinoma. Front Oncol.

[76] Przybylski K, Majchrzak E, Weselik L, Golusiński W (2018). Immunotherapy of head and neck squamous cell carcinoma (HNSCC). Immune checkpoint blockade. Otolaryngol Pol.

[77] Bhatia A, Burtness B (2023). Treating head and neck cancer in the age of immunotherapy: a 2023 update. Drugs.

[78] Ge J, Wang J, Xiong F (2021). Epstein-barr virus-encoded circular RNA circBART2.2 promotes immune escape of nasopharyngeal carcinoma by regulating PD-L1. Cancer Res.

[79] Zhang J, Hu H, Zhao Y, Zhao Y (2018). CDR1as is overexpressed in laryngeal squamous cell carcinoma to promote the tumour’s progression via miR-7 signals. Cell Prolif.

[80] Chen Y, Li Z, Liang J (2022). CircRNA has_circ_0069313 induced OSCC immunity escape by miR-325-3p-Foxp3 axes in both OSCC cells and Treg cells. Aging.

[81] (2019). Burtness B, Harrington KJ, Greil R, et al; KEYNOTE-048 Investigators. Pembrolizumab alone or with chemotherapy versus cetuximab with chemotherapy for recurrent or metastatic squamous cell carcinoma of the head and neck (KEYNOTE-048): a randomised, open-label, phase 3 study. Lancet.

[82] (2019). Cohen EEW, Soulières D, Le Tourneau C, et al; KEYNOTE-040 investigators. Pembrolizumab versus methotrexate, docetaxel, or cetuximab for recurrent or metastatic head-and-neck squamous cell carcinoma (KEYNOTE-040): a randomised, open-label, phase 3 study. Lancet.

[83] Han J, Zakeri K, Raab G (2023). Concurrent carboplatin and paclitaxel definitive radiation therapy for locally advanced head and neck cancer. Head Neck.

[84] Alexa-Stratulat T, Pešić M, Gašparović AČ, Trougakos IP, Riganti C (2019). What sustains the multidrug resistance phenotype beyond ABC efflux transporters? Looking beyond the tip of the iceberg. Drug Resist Updat.

[85] Vasan N, Baselga J, Hyman DM (2019). A view on drug resistance in cancer. Nature.

[86] Huang A, Zheng H, Wu Z, Chen M, Huang Y (2020). Circular RNA-protein interactions: functions, mechanisms, and identification. Theranostics.

[87] Hong X, Liu N, Liang Y (2020). Circular RNA CRIM1 functions as a ceRNA to promote nasopharyngeal carcinoma metastasis and docetaxel chemoresistance through upregulating FOXQ1. Mol Cancer.

[88] Liu L, Lu B, Li Y (2022). Circular RNA circ_0008450 regulates the proliferation, migration, invasion, apoptosis and chemosensitivity of CDDP-resistant nasopharyngeal carcinoma cells by the miR-338-3p/SMAD5 axis. Anticancer Drugs.

[89] Hong X, Li Q, Li J (2022). CircIPO7 promotes nasopharyngeal carcinoma metastasis and cisplatin chemoresistance by facilitating YBX1 nuclear localization. Clin Cancer Res.

[90] Cheng Y, Zhu Y, Xiao M (2023). circRNA_0067717 promotes paclitaxel resistance in nasopharyngeal carcinoma by acting as a scaffold for TRIM41 and p53. Cell Oncol.

[91] Ai J, Tan G, Li W (2023). Exosomes loaded with circPARD3 promotes EBV-miR-BART4-induced stemness and cisplatin resistance in nasopharyngeal carcinoma side population cells through the miR-579-3p/SIRT1/SSRP1 axis. Cell Biol Toxicol.

[92] Qiongna D, Jiafeng Z, Yalin H (2020). Implication of hsa_circ_0028007 in reinforcing migration, invasion, and chemo-tolerance of nasopharyngeal carcinoma cells. J Clin Lab Anal.

[93] Lin J, Qin H, Han Y, Li X, Zhao Y, Zhai G (2021). CircNRIP1 modulates the miR-515-5p/IL-25 axis to control 5-Fu and cisplatin resistance in nasopharyngeal carcinoma. Drug Des Devel Ther.

[94] Feng B, Chen K, Zhang W, Zheng Q, He Y (2021). circPGAM1 enhances autophagy signaling during laryngocarcinoma drug resistance by regulating miR-376a. Biochem Biophys Res Commun.

[95] Yi X, Chen W, Li C (2021). Circular RNA circ_0004507 contributes to laryngeal cancer progression and cisplatin resistance by sponging miR-873 to upregulate multidrug resistance 1 and multidrug resistance protein 1. Head Neck.

[96] Wu S, Lv X, Wei H (2023). Circ-ILF2 in oral squamous cell carcinoma promotes cisplatin resistance and induces M2 polarization of macrophages. J Cell Mol Med.

[97] (2023). Ren W, Cheng Y, Li S, Zheng J, Gao L, Zhi K. circAP1M2 activates ATG9A-associated autophagy by inhibiting miR-1249-3p to promote cisplatin resistance in oral squamous cell carcinoma. J Cell Physiol.

[98] Gao F, Han J, Wang Y, Jia L, Luo W, Zeng Y (2022). Circ_0109291 promotes cisplatin resistance of oral squamous cell carcinoma by sponging miR-188-3p to increase ABCB1 expression. Cancer Biother Radiopharm.

[99] Yan J, Xu H (2021). Regulation of transforming growth factor-beta1 by circANKS1B/miR-515-5p affects the metastatic potential and cisplatin resistance in oral squamous cell carcinoma. Bioengineered.

[100] Su M, Xiao Y, Ma J (2019). Circular RNAs in Cancer: emerging functions in hallmarks, stemness, resistance and roles as potential biomarkers. Mol Cancer.

